# Genetic and functional characterization of a novel *SERPIND1* variant in a family with isolated gastric varices and venous thrombosis

**DOI:** 10.1186/s12876-026-05163-z

**Published:** 2026-07-23

**Authors:** Xue-yan Lin, Li-jin Zhang, Jian-hui Zhang, Ruo-li Wang, Li-fang Lin, Gui-fang Huang, Dan-dan Ruan, Zhi-hai Zheng, Jie-wei Luo, Ming-hong Chen

**Affiliations:** 1 Department of Gastroenterology and Hepatology and Traditional Chinese Medicine and Nephrology, Shengli Clinical Medical College of Fujian Medical University, Fuzhou University Affiliated Provincial Hospital, No. 134, Dongjie, Fuzhou, 350001 China; 2 Fujian Provincial Hospital, Fuzhou University Affiliated Provincial Hospital, Fuzhou University, Fuzhou, China

**Keywords:** *SERPIND1* gene, *KCNH2* gene, Heparin cofactor Ⅱ, Isolated gastric varices, Long QT syndrome type 2, Thrombophilia

## Abstract

**Background:**

Isolated gastric varices type I (IGV1) is a rare but severe manifestation of splenic vein thrombosis. While thrombophilia is a recognized cause, the underlying genetic determinants are often elusive. This study investigates the case of a proband presenting with IGV1, deep vein thrombosis (DVT), and Long QT syndrome Type 2 (LQT2), aiming to elucidate the genetic basis and functional impact of the identified *SERPIND1* variants.

**Methods:**

Whole-exome sequencing identified candidate variants in the proband, which were validated by Sanger sequencing. Functional assessment included quantifying *SERPIND1* and *KCNH2* mRNA in proband and control blood, analyzing heparin cofactor Ⅱ (HCⅡ) protein expression and localization in transfected 293T cells, and predicting the potential structural consequences of the variants using bioinformatic tools.

**Results:**

The proband, a 31-year-old female, presented with isolated gastric fundal varices and a history of deep venous thrombosis (DVT), along with long QT syndrome type 2 (LQT2). Whole-exome sequencing (WES) identified two heterozygous variants, both confirmed by Sanger sequencing: a de novo *SERPIND1* c.940 C > T (p.Arg314Trp) variant, classified as Pathogenic according to ACMG guidelines (PS2, PS3, PM2_Supporting, PP3), and a *KCNH2* c.1707 C > A (p.Tyr569*) variant, also classified as Pathogenic (PVS1, PP1, PM2_Supporting) and showing familial co-segregation. The *SERPIND1* variant was associated with reduced HCⅡ antigen levels and activity in plasma. In vitro assays revealed that both variants led to significantly reduced *SERPIND1* and *KCNH2* mRNA expression. In transfected cells, the *SERPIND1* p.Arg314Trp variant markedly decreased HCⅡ protein levels and possibly induced aberrant nuclear mislocalization. Structural modeling suggested that the Arg314Trp substitution disrupts a key hydrogen bond within HCⅡ, while the *KCNH2* p.Tyr569* nonsense variant is predicted to generate a truncated protein.

**Conclusion:**

The *SERPIND1* c.940 C > T (p.Arg314Trp) variant is associated with reduced HCⅡ expression and functional impairment, which may contribute to a prothrombotic tendency in this patient. This study provides a hypothesis-generating observation supporting a potential role of HCⅡ deficiency in venous thrombosis and IGV1. The *KCNH2* variant was accounted for the coexisting LQT2 phenotype.

**Supplementary Information:**

The online version contains supplementary material available at https://doi.org/10.1186/s12876-026-05163-z.

## Introduction

Isolated gastric varices (IGV) refer to gastric varices that occur without accompanying esophageal varices. They are classified into two types: Isolated Gastric Varices Type 1 (IGV1), located in the gastric fundus, and Ectopic Isolated Gastric Varices (IGV2), found in other regions of the stomach. IGV1 is relatively rare, with an estimated prevalence of approximately 5–10% among all patients with gastric varices, but is associated with more severe bleeding and a higher risk of mortality compared to gastroesophageal varices (GOV) [1, 2]. Studies have shown that gastric varices are more commonly observed in regional portal hypertension (RPH) than in cirrhosis-induced portal hypertension [3, 4]. The underlying cause of RPH is splenic vein obstruction, most frequently due to acute or chronic pancreatitis or pancreatic tumor [5]. In contrast, splenic vein occlusion caused by multiple thromboses is rarely reported, with only isolated case reports [6, 7].

Hereditary deficiencies of antithrombin Ⅲ (ATⅢ), protein C, protein S, heparin cofactor Ⅱ (HCⅡ) and essential thrombocythemia (ET) account for 10–15% of recurrent venous thrombosis cases [8, 9]. Splenic vein thrombosis-induced IGV1 secondary to protein S deficiency, antiphospholipid syndrome (APS), or ET has only been sporadically reported [6, 7]. While, the thrombotic risk associated with HCⅡ deficiency is not well established, and no cases of HCⅡ deficiency-induced splenic vein thrombosis complicated by IGV1 have been documented.

Encoded by the *SERPIND1* gene (NM_000185.4), HCⅡ is a serine protease inhibitor first cloned from liver tissue by Blinder et al. [10] and Ragg [11] in 1988. Mapped to chromosome 22, its 480-residue mature polypeptide is preceded by a 19-residue signal peptide. Functionally, As a unique member of the serpin family, HCⅡ is a 65.5 kDa plasma glycoprotein that inhibits thrombin through a mechanism requiring glycosaminoglycans like dermatan sulfate or heparin to amplify its anticoagulant activity [12]. In its native resting state, the reactive center loop (RCL) of HCⅡ is concealed within the protein’s structure, preventing efficient interaction with thrombin [13]. Conformational activation of HCⅡ occurs primarily through binding to dermatan sulfate (DS), a glycosaminoglycan (GAG) enriched in the vascular wall, as well as heparin or heparan sulfate (HS) with lower affinity under physiological conditions. Experimental studies also demonstrate activation by synthetic polyanions (e.g., polyphosphates) via electrostatic interactions [14–17]. DS binding triggers a structural rearrangement that releases the reactive center loop (RCL) from its sequestered position. The exposed RCL acts as a bait, enabling HCⅡ to form a 1:1 covalent complex with thrombin via cleavage at Arg444-Thr445. This interaction traps thrombin through insertion of the RCL into β-sheet A, irreversibly inactivating the protease via the canonical serpin mechanism [13, 18, 19].

*KCNH2* gene (NM_000238.4) encodes the K_v_11.1 protein, the α-subunit of the potassium channel mediating the rapid delayed rectifier potassium current (I_Kr_), which is essential for cardiomyocyte repolarization and neuronal excitability control [20]. Loss-of-function (LOF) mutations in *KCNH2* reduce I_Kr_, leading to prolonged cardiac action potentials and increased risk of arrhythmias [21, 22]. Clinically, such mutations are associated with Long QT Syndrome Type 2 (LQT2), characterized by QT interval prolongation and a predisposition to ventricular arrhythmias and sudden cardiac death, and LQT2 affects approximately 1 in 2,000–3,000 individuals worldwide [23].

We describe a rare case of a *SERPIND1* variant associated with IGV1 and multi-territory venous thrombosis. Functional analyses demonstrated that the *SERPIND1* c.940 C > T (p.Arg314Trp) variant is associated with reduced HCⅡ expression and abnormal subcellular localization, consistent with a loss-of-function effect and a prothrombotic tendency. In addition, a concurrent *KCNH2* c.1707 C > A (p.Tyr569*) nonsense mutation was identified and is responsible for the LQT2 phenotype in this family. This finding is interpreted as an independent and unrelated genetic diagnosis. No mechanistic or clinical evidence supports an association between *KCNH2* dysfunction and venous thrombosis in this case. Therefore, *KCNH2* is not considered to contribute to the thrombotic phenotype.

## Materials and methods

### Research subjects

This study conducted a systematic evaluation of a thrombophilic pedigree from Fujian, China, in which the proband (II4) presented with IGV1. The pedigree included 11 members. Although the proband’s eldest daughter (III3) carries two pathogenic genetic mutations identical to the proband, but exhibits no clinical manifestations similar to the proband. Additionally, the other nine family members (I1, I2, II1, II2, II3, II5, III1, III2, III4) showed no history of thrombosis or gastrointestinal bleeding and were designated as the healthy control group. Relevant medical history and clinical data were collected from family members, including imaging studies and coagulation parameters. This study was approved by the Ethics Committee of Fuzhou University Affiliated Provincial Hospital, and written informed consent was obtained from the proband and all participating family members.

### Clinical phenotype detection

Clinical phenotypes and biochemical laboratory indices were collected from the proband and her family members. Biochemical testing included: Protein S (PS) activity, measured by a clotting method (STA-Liquid Anti-Total PS, Stago); Protein C (PC) and AT-III activities, measured by chromogenic substrate methods (Berichrom^®^ PC/AT, Siemens); Anti-phosphatidylserine/prothrombin (aPS/PT) IgG/IgM antibodies, detected by chemiluminescent immunoassay (QUANTA Flash^®^ aPS/PT, Inova Diagnostics); Plasma HCⅡ antigen levels were measured using a human HCⅡ ELISA kit (Beijing Jinyu Bio, China; Cat. No. JY-HCII-01), and HCⅡ functional activity was assessed using a thrombin inhibition-based chromogenic assay in the presence of dermatan sulfate and heparin (Beijing Jinyu Bio, China; Cat. No. JY-HCII-A01). All assays were performed by KingMed Diagnostics (Guangzhou, China) according to the manufacturer’s protocols.

### Extraction of genomic DNA

Peripheral blood samples were collected using ethylenediaminetetraacetic acid (EDTA) anticoagulant tubes. Peripheral blood mononuclear cell DNA was extracted using the QIAamp DNA Blood Mini Kit (QIAGEN, DP705) according to the manufacturer’s instructions. The purified DNA samples were used for sequencing.

### Genetic analysis and variant screening

Genomic DNA was extracted from peripheral blood and subjected to whole-exome sequencing (WES). DNA libraries were prepared using the NadPrep DNA Fast Enzymatic Library Preparation Kit (MGI-tech, China) following fragmentation, end repair, adapter ligation, and PCR amplification. The PCR products were purified with NadPrep^®^ SP Beads (MGI-tech, China). Library quality was assessed using Nanodrop and Qubit 3.0 systems. Target enrichment was performed using the IDT xGen Exome Research Panel v1.0 (Integrated DNA Technologies, USA), and sequencing was conducted on the GeneMind SURFSeq5000 platform (GeneMind, China). Variant calling was performed using the Genome Analysis Toolkit (GATK). Based on the proband’s clinical features, phenotype terms were curated using the Human Phenotype Ontology (HPO) and integrated with the sequencing data for analysis in Exomiser to prioritize candidate variants. Variants were filtered by excluding those with a minor allele frequency (MAF) > 1% in the ExAC or gnomAD databases, or > 5% in the iGeneTechDB local database. iGeneTechDB is a proprietary in-house database containing exome sequencing data from over 10,000 individuals of predominantly East Asian ancestry. It is a mature and reliable resource, which was used here to exclude common population variants and aid in candidate variant prioritization. Synonymous variants without predicted splicing impact and variants classified as benign/likely benign in ClinVar were also excluded. A final candidate list was generated by cross-referencing with a curated list of disease-related genes.

Suspicious variants were screened and interpreted according to the guidelines of the American College of Medical Genetics and Genomics (ACMG). Specific primers targeting the upstream and downstream regions of the mutation sites were designed using Primer 5.0, and the corresponding target regions were amplified. Sanger sequencing validation was performed using an ABI 3730XL DNA sequencer (Applied Biosystems, USA). For the *SERPIND1* variant site at chr22:21138310, c.940 C > T (p.Arg314Trp), the amplified fragment length was 250 bp, with the following primers:

Forward (F): ACCTTCTCATAACAGCCTCTTCC, Reverse (R): TCTTCATCCCAGACATCTTGTG. The annealing temperature was 57 °C. For the *KCNH2* mutation site at chr7:150648774, c.1707 C > A (p.Tyr569*), the amplified fragment length was 245 bp, with the following primers: Forward (F): ATCAACGGAATGTGCCCC, Reverse (R): GGGTTTGCCTATCTGGTCG. The annealing temperature was 59 °C. A pedigree-based genetic and phenotypic investigation was conducted to verify the presence of co-segregation within the family.

### Construction and validation of wild-type and p.Arg314Trp mutant *SERPIND1* plasmids

The coding sequence of *SERPIND1* was synthesized and cloned into the PCDH-CMV-HA-EF1-GFP-T2A-Puro vector using *KpnI* and *XhoI* restriction sites, resulting in two constructs: a wild-type plasmid (PCDH-CMV-HA-*SERPIND1* WT-EF1-GFP-T2A-Puro) and a mutant plasmid (PCDH-CMV-HA-*SERPIND1* MUT-EF1-GFP-T2A-Puro) carrying the pathogenic c.940 C > T variant (p.Arg314Trp). The target sequence was amplified for verification. Gene cloning and the synthesis of related PCR primers were performed by Wuhan GeneCreate Biological Engineering Co., Ltd.

### Cell transfection

293T cells were trypsinized and seeded in 24-well plates at a density of 0.5–2 × 10^5^ cells per plate in complete medium. At 60–70% confluency, cells were transfected using Lipofectamine^®^ 3000 (Thermo, L3000015). Per well, 20 pmol plasmid DNA was mixed with 25 µL Opti-MEM (Gibco, BS350B) and 1.5 µL Lipofectamine^®^ 3000. After 5min incubation at room temperature, complexes were added dropwise to cells, and incubated at 37 °C in 5% CO₂. Medium was replaced with serum-containing medium after 8–12 h.

### Reverse transcriptase-quantitative (RT-qPCR) analysis

Total RNA was extracted from peripheral blood samples of the proband and healthy controls using TRIpure Isolation Reagent (Roche, 11667165001). RNA was reverse transcribed into first-strand cDNA using the RevertAid First Strand cDNA Synthesis Kit (Thermo, K1691), according to the manufacturer’s instructions. The 20 µL reverse transcription reaction system included 4 µL of 5× RT Mix, 1 µg of RNA template, 1 µL of Oligo(dT) and Random Primer Mix, and nuclease-free water to volume. The reaction was incubated at 37 °C for 15 min and 85 °C for 5 s to inactivate the reverse transcriptase. The resulting cDNA was diluted 20-fold with nuclease-free water and stored at -20 °C until use. Quantitative PCR (qPCR) was subsequently performed using a SYBR Green qPCR Master Mix (Roche, 25325100) on a CFX96 Real-Time PCR System (Bio-Rad). The primer sequences used are listed in Table 1.

**Table 1:** Primers for RT-qPCR

Primer name	Primer sequence
SERPIND1-1 F (preceding the mutation site)	CGGATCCAGCGTCTTAACAT
SERPIND1-1R (preceding the mutation site)	TGCTGCTGGCATTAACAAAG
SERPIND1-2 F (following the mutation site)	AATGACCAGGAGCTGGACTG
SERPIND1-2R (following the mutation site)	TCCTGATCCCCATCAACTTC
KCNH2-1 F (preceding the mutation site)	CTCAACTCCACCTCGGACTC
KCNH2-1R (preceding the mutation site)	TTGTACTCAGGCAGCACGTC
KCNH2-2 F (following the mutation site)	CTCACCGCCCTGTACTTCAT
KCNH2-2R (following the mutation site)	GACCAGAAGTGGTCGGAGAA
GAPDH-F	GGAGCGAGATCCCTCCAAAAT
GAPDH-R	GGCTGTTGTCATACTTCTCATGG

### Western blotting

The expression of recombinant HCⅡ was analyzed by Western blot using an HA-tag-based detection strategy. 293T cells were transfected with HA-tagged *SERPIND1* wild-type or mutant constructs. Total protein was extracted from the transfected cells, separated by SDS-PAGE, and electrophoretically transferred to a PVDF membrane. The membrane was blocked with 5% BSA and subsequently probed with an anti-HA primary antibody (anti-HA tag, Abclonal, AE008, 1:1000; anti-GAPDH, Abclonal, AC002, 1:1000) overnight at 4 °C. After washing, the membrane was incubated with an HRP-conjugated secondary antibody (Goat anti Rabbit IgG, Abcam, ab6721, 1:2000). Protein bands were visualized using an enhanced chemiluminescence (ECL) detection system.

### Immunofluorescence localization assay

293T cells were fixed, permeabilized, and blocked after transfection. The primary antibody (anti-HA tag, Abclonal, AE008, 1:200) was incubated overnight at 4 °C. After washing five times with PBS, fluorescent secondary antibody (Alexa Flour 549 chicken anti-mouse IgG(H + L), Invitrogen, A21201) was added and incubated for 1 h at room temperature in the dark. The cells were then washed again with PBS five times. A total of 5 µL of antifade mounting medium containing DAPI (Beyotime, P0131) was placed on a microscope slide, and the coverslip with the cells was inverted onto it. After sealing, images were captured using a laser confocal microscope (Nikon Tis, Japan).

### Protein structures of *SERPIND1* c.940 C > T and *KCNH2* c.1707 C > A variant

The three-dimensional (3D) structures of both the wild-type (WT) and mutant forms of the human HCⅡ/HEP2 protein (UniProt ID: P05546) and the human Ether-à-go-go-Related Gene (hERG) potassium channel protein (UniProt ID: Q12809) were predicted using the Swiss-Model server (https://swissmodel.expasy.org/repository/uniprot/P05546; https://swissmodel.expasy.org/repository/uniprot/Q12809). Visualization using PyMOL revealed structural alterations in the proteins resulting from the variants.

### Statistical analysis

Experimental data were analyzed using GraphPad Prism 7.0. Comparisons between two groups were performed using unpaired Student’s t-tests. Data are expressed as mean ± SEM. A p-value < 0.05 was considered statistically significant.

## Results

### Clinical phenotype

The proband (II4) is a 31-year-old female who presented with a 6-day history of hematemesis and melena. This episode occurred two years after the birth of her second child. Five years prior, during the puerperium following her first delivery, she developed swelling of the left lower extremity and was diagnosed with deep vein thrombosis (DVT) involving the left superficial femoral vein, left iliac vein, and inferior vena cava (IVC). She underwent one session of thrombolytic therapy followed by one year of oral rivaroxaban anticoagulation. Follow-up imaging confirmed resolution of the thrombus, after which anticoagulation was discontinued, and no further monitoring was performed. During her second pregnancy, she was administered low-molecular-weight heparin (LMWH), which was discontinued immediately after delivery without subsequent follow-up. She reported no history of liver or pancreatic disease and denied unhealthy lifestyle habits such as smoking or alcohol consumption.

**Fig. 1:**
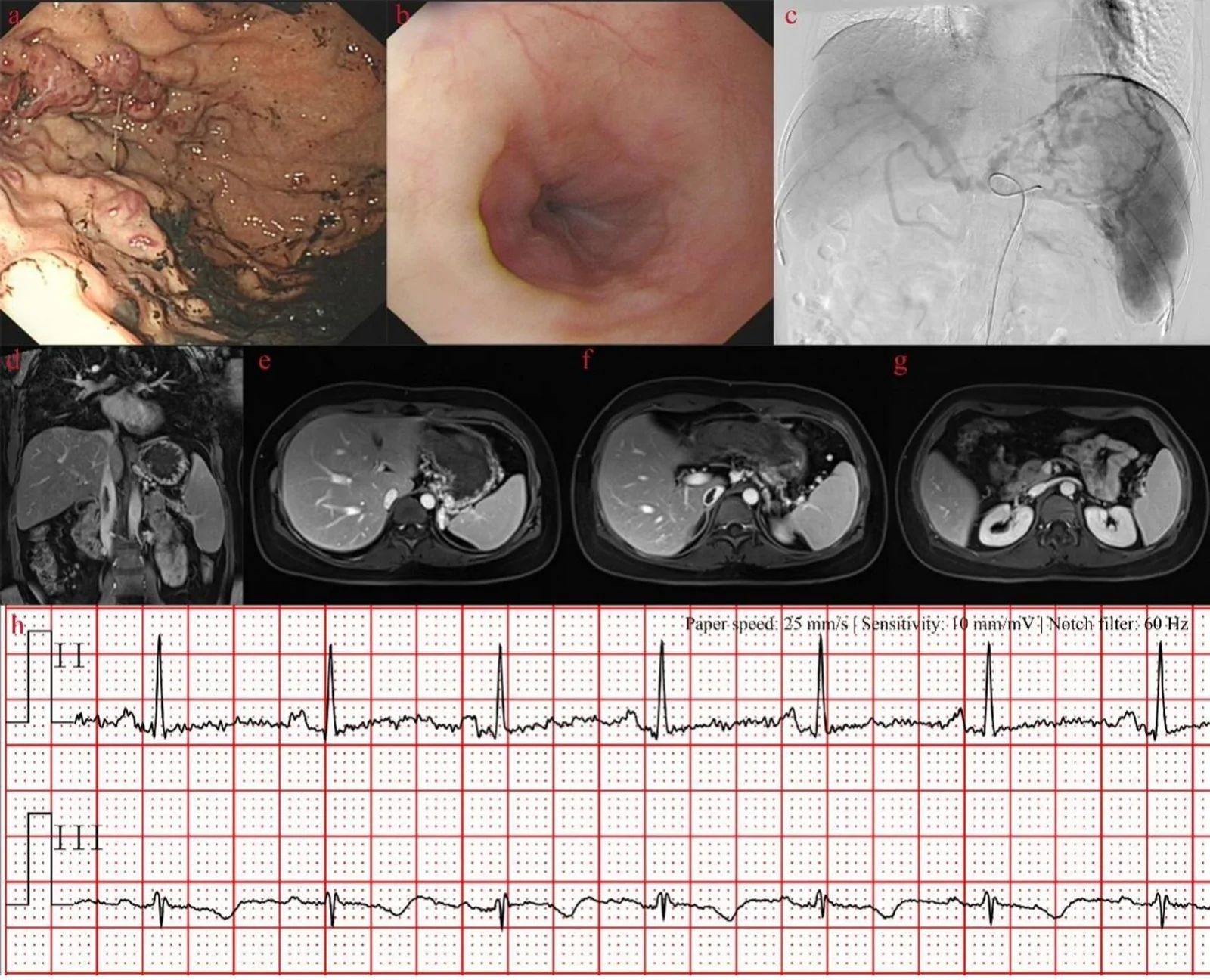
Multimodal imaging of the proband. **a**-**b** Esophagogastroduodenoscopy showing isolated gastric fundal varices without esophageal involvement. **c** Digital subtraction angiography of abdominal vasculature revealing complete splenic vein occlusion. **d**-**g** Axial and coronal views of contrast-enhanced upper abdominal MRI demonstrating a normal liver and spleen, with thrombus in the inferior vena cava and thrombus extension into the right renal vein, gastric fundal and splenic vein varices. **h** The electrocardiogram showed a prolonged QTc interval of 480 milliseconds

During the current hospitalization, comprehensive diagnostic evaluations were conducted. Esophagogastroduodenoscopy (EGD) revealed IGV1 (Fig. 1a and b), contrast-enhanced MRI of the liver demonstrated no evidence of cirrhosis or splenomegaly but identified thrombosis in the inferior vena cava (IVC), along with varices in the gastric fundal and splenic veins (Fig. 1d, e, f and g). Digital subtraction angiography (DSA) confirmed splenic vein occlusion and thrombosis involving the right renal vein and IVC (Fig. 1c). The electrocardiography (ECG) shows a QTc interval of 480 milliseconds (Fig. 1h), and transthoracic echocardiography showed no structural abnormalities. Laboratory investigations showed normal prothrombin time (PT), ATⅢ, protein S (PS), and protein C (PC) levels (Table 2). In addition, tests for anti-β2-glycoprotein antibodies, anti-phosphatidylserine/prothrombin antibodies, antinuclear antibodies (ANA), anti-neutrophil cytoplasmic antibodies (ANCA), lupus anticoagulant (LA1 and LA2), and anticardiolipin antibodies (IgG and IgM) were all negative (Supplementary Table 1). In addition, to further assess the proband’s anticoagulant profile, plasma HCⅡ antigen levels and activity were measured. Results demonstrated a reduction in both HCⅡ antigen concentration and functional activity compared with reference ranges, supporting a clinically relevant HCⅡ deficiency in this patient (Supplementary Table 1).

**Table 2:** Blood coagulation function indexes of proband and family members

Test item	Proband (II4)	Proband father(I1)	Proband mother(I2)	Member (II1)	Member (II2)	Member (III3)	Member (III4)	Reference value
APTT (sec)	24.9	25.0	26.2	25.2	25.4	25.1	25.6	20.0-45.9
PT (sec)	12.3	11.2	11.8	11.3	11.4	11.2	11.6	9.7–15.7
TT (sec)	16.1	15.1	16.8	15.3	15.5	15.2	15.8	12.3–22.0
Fg (g/L)	2.8	2.5	3.2	2.6	2.6	3.2	3.1	2.0–4.0
FDP (mg/L)	4.0	3.8	3.2	3.4	3.3	3.8	2.9	0–5.0
D-dimer (mg/L)	1.49	0.14	0.02	0.08	0.11	0.84	0.05	0-0.55
INR	1.07	0.92	1.08	0.93	0.94	1.06	0.96	
AT: A (%)	80.7	90.2	96.3	91.2	91.5	86.8	100.2	75.0-125.0
PS (%)	70.8	63.2	71.1	/	/	80.1	/	60.0-130.0
PC (%)	111.1	120.7	134.0	/	/	130.0	/	70.0-150.0
Anti-phosphatidylserine/prothrombin IgG antibody (U/mL)	6.65	5.51	5.03	/	/	5.93	/	<30.0
Anti-hosphatidylserine/prothrombin IgM antibody (U/mL)	29.2	23.4	20.8	/	/	26.6	/	<30.0

*APTT* Activated partial thromboplastin time, *PT* Prothrombin time, *TT* Thrombin time, *Fg* Fibrinogen, *FDP* Fibrin degradation products, *INR* International normalized ratio, *PS* Protein S, *PC* Protein C, *AT* A Antithrombin Activity

Following confirmation of the diagnosis, the patient underwent laparoscopic splenectomy combined with devascularization of gastric fundal varices under general anesthesia on March 7, 2025. The postoperative course was uneventful, with no significant complications. Long-term anticoagulation with rivaroxaban was initiated postoperatively. The follow-up plan included outpatient monitoring of coagulation parameters every 1–3 months and contrast-enhanced abdominal CT every 3–6 months to assess for recurrent thrombosis. The patient had no symptoms such as palpitations, chest tightness, or syncope; therefore, no specific antiarrhythmic treatment was initiated. She was advised to undergo electrocardiographic follow-up every 6 months and to avoid medications known to prolong the QT interval.

**Fig. 2:**
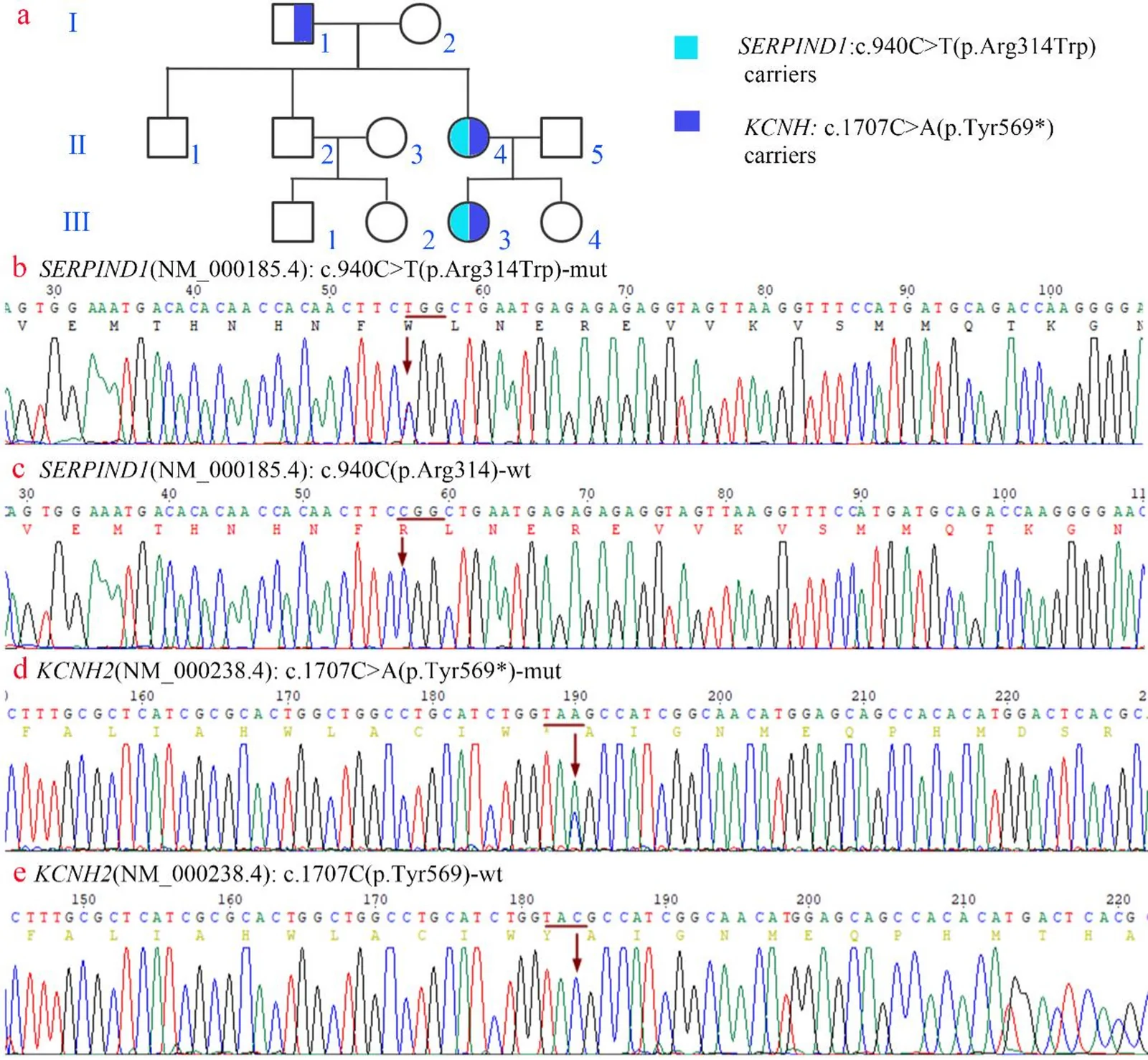
Pedigree and sanger sequencing of a family with *SERPIND1* and *KCNH2* gene variants. **a** Circles and squares represent females and males, respectively. The II4 indicates the proband. Light and dark blue shading denote carriers of the heterozygous *SERPIND1* c.940 C > T and *KCNH2* c.1707 C > A variants, respectively. The proband is a double heterozygote. **b–c** Sanger sequencing chromatograms showing the *SERPIND1* c.940C>T (p.Arg314Trp) variant (**b**) and the wild-type sequence (**c**). **d–e** Sanger sequencing chromatograms showing the *KCNH2* c.1707C>A (p.Tyr569*) variant (**d**) and the wild-type sequence (**e**)

Patient III3, an 8-year-old female, had no prior history of lower limb swelling or pain. However, recent evaluation of the deep and superficial veins of the lower extremities revealed thrombosis of the right common femoral vein, for which rivaroxaban anticoagulation was subsequently initiated. No thrombotic events were identified in the other family members. Supplementary Table 3 summarizes the genotype–phenotype correlation and clinical characteristics of the family members. Based on the above clinical data, a genetic pedigree of this family was constructed (Fig. 2a).

### Genetic screening for thrombophilia-associated mutations

This whole-exome sequencing (WES) analysis was performed by targeted capture of exonic regions from patient genomic DNA followed by high-depth next-generation sequencing. The assay achieved 99.9% coverage at ≥ 1× and 99.8% coverage at ≥ 10× across the target regions, with a mean sequencing depth of 100×. Sequencing quality metrics demonstrated that ≥ 85% of bases reached a Q30 quality score and ≥ 90% reached Q20, indicating high data quality suitable for downstream variant analysis. WES identified two heterozygous variants in the proband associated with thrombotic and LQT2. One was a *SERPIND1* (NM_000185.4) c.940 C > T (p.Arg314Trp) variant in exon 3, which was not detected in either parent, nor was it identified in any earlier-generation family members, supporting its de novo origin. With a very low population frequency and supported by our in vitro functional evidence indicating loss of function, this variant was classified as pathogenic according to ACMG guidelines (PS2, PS3, PM2_Supporting, PP3). The other was a *KCNH2* (NM_000238.4) c.1707 C > A (p.Tyr569*) nonsense variant in exon 7, which co-segregated with the disease in the family, was inherited from the father, and was detected in affected members (I1, II4, and III3) diagnosed with LQT2. It was also classified as pathogenic (PVS1, PP1, PM2_Supporting). Sanger sequencing confirmed that nucleotide 940 in exon 3 of the *SERPIND1* gene was substituted from cytosine (C) to thymine (T), resulting in an arginine-to-tryptophan substitution (Fig. 2b). Similarly, nucleotide 1707 in exon 7 of the *KCNH2* gene was changed from cytosine (C) to adenine (A), introducing a premature stop codon and leading to a truncated protein (Fig. 2d). The proband’s elder daughter (III3) carried both heterozygous variants, and ultrasonography revealed deep vein thrombosis of the lower limbs.

### Cell transfection status

**Fig. 3:**
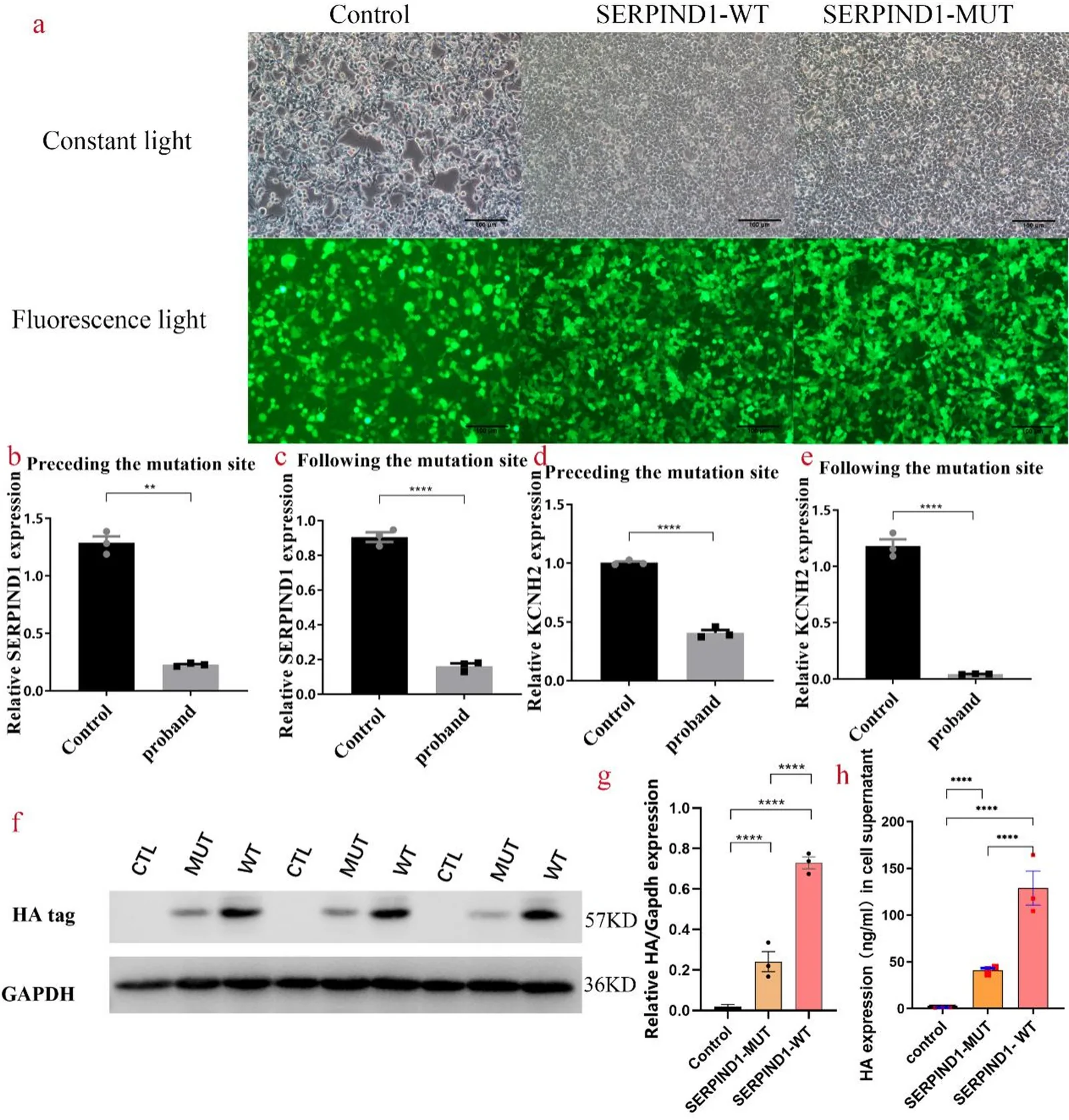
Functional validation of the *SERPIND1* and *KCNH2* variants. **a** Functional analysis of *SERPIND1* mutant on gene expression and HCⅡ protein levels. Cell morphology of 293T cells 24 h after transfection with empty vector (PCDH), wild-type (SERPIND1-WT), and p.Arg314Trp mutant (SERPIND1-MUT). **b**-**c** q-PCR analysis of *SERPIND1* mRNA expression in healthy controls and the proband. **d**-**e** q-PCR analysis of KCNH2 mRNA expression in healthy controls and the proband. **f** Western blot analysis of HCⅡ expression in 293T cell lysates transfected with empty vector (CTL), *SERPIND1* mutant (MUT), and *SERPIND1* wild-type (WT). **g** The relative expression of HA (HCⅡ) in 293T cell lysates analyzed by Image J. **h** HA (HCⅡ) expression in the supernatant of transfected 293T cells. Statistical analysis was performed using unpaired Student’s t-tests between groups, with *p* < 0.05 considered statistically significant. **p < 0.01, ****p < 0.0001

To investigate the functional consequences of the *SERPIND1* variant, we performed a series of assays in transfected 293T cells. The cloning vectors for *SERPIND1* wild-type (SERPIND1-WT) and the p.Arg314Trp mutant (SERPIND1-MUT) were successfully constructed. Empty vector (PCDH), SERPIND1-WT vector, and SERPIND1-MUT vector were transfected into 293T cells, respectively. As shown in Fig. 3a, 24 h post-transfection, all groups displayed normal cell morphology and good viability, indicating that transfection did not adversely affect cell growth.

### Impact of *SERPIND1* variant on mRNA and HCⅡ protein expression

We then assessed the impact of the variant on gene expression. To comprehensively evaluate the *SERPIND1* mRNA levels, we designed two specific primer pairs for q-PCR analysis: one pair located upstream and the other downstream of the variant site. Using both primer sets, we consistently confirmed a significant downregulation of *SERPIND1* mRNA in the proband compared to healthy controls (Fig. 3b, c). Intriguingly, a similar analysis of *KCNH2* expression, also conducted with two independent primer pairs, revealed a similarly and marked decrease in its mRNA levels in the proband (Fig. 3d, e).

To determine whether the reduced mRNA level translated to a defect at the protein level, we analyzed HCⅡ expression by Western blotting. The results demonstrated that HCⅡ protein expression was decreased in cells transfected with the SERPIND1-MUT compared with cells transfected with SERPIND1-WT or empty vector (Fig. 3f). Quantitative analysis using ImageJ confirmed a significant reduction in HCⅡ protein levels in the MUT group (**p < 0.01, ****p < 0.0001; Fig. 3g). Detailed gray value calculation results are shown in Supplementary Table 2. At the same time, HCⅡ protein levels in the culture supernatants were measured by ELISA (Fig. 3h). The results showed that cells transfected with the *SERPIND1* p.Arg314Trp mutant exhibited significantly lower HCⅡ protein secretion compared to the WT group, consistent with the Western blot findings. These results indicate that the p.Arg314Trp variant in *SERPIND1* impairs HCⅡ protein expression in 293T cells.

### Cellular localization of wild-type and mutant *SERPIND1*

**Fig. 4:**
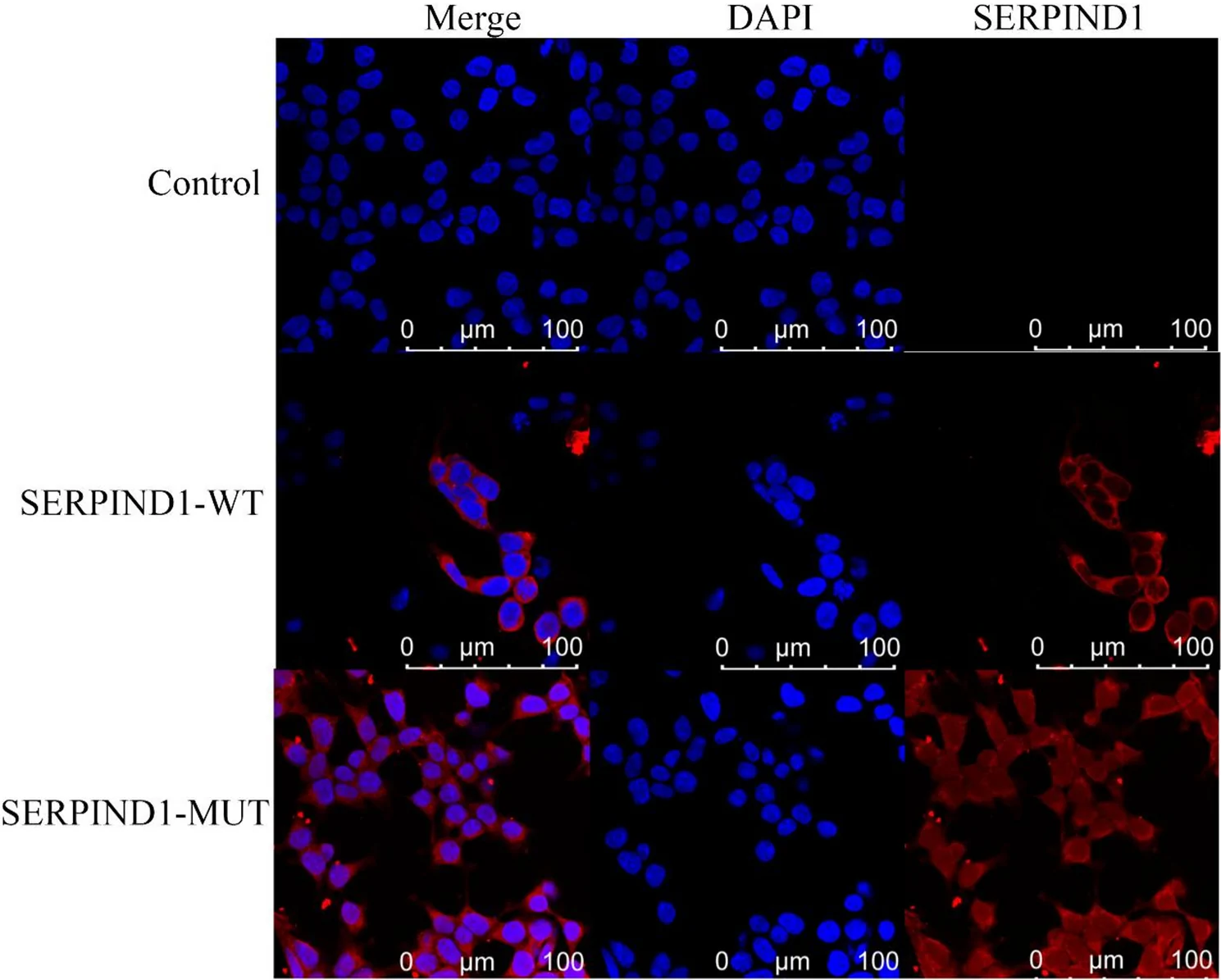
Subcellular mislocalization of mutant HCⅡ protein. Altered subcellular localization of mutant HCⅡ protein. Immunofluorescence analysis of 293T cells expressing empty vector (PCDH), wild-type (SERPIND1-WT), and p.Arg314Trp mutant (SERPIND1-MUT) shows that the wild-type protein was predominantly localized in the cytoplasm with only faint nuclear signals, whereas the mutant protein retained cytoplasmic localization but accumulated extensively within the nucleus

To investigate whether the *SERPIND1* p.Arg314Trp variant affects HCⅡ protein localization, 293T cells were transfected with empty vector (PCDH), wild-type (SERPIND1-WT), or mutant (SERPIND1-MUT), and analyzed by immunofluorescence. Immunofluorescence analysis demonstrated that the introduced variant significantly altered the subcellular distribution of HCⅡ (Fig. 4). While the wild-type protein was predominantly cytoplasmic with negligible nuclear signal, the mutant variant displayed a pronounced redistribution, accumulating substantially within the nucleus in addition to its cytoplasmic presence. This shift from a cytoplasm-dominant to a dual cytoplasmic/nuclear localization indicates disrupted nucleocytoplasmic trafficking.

Notably, this aberrant localization coincided with an overall reduction in HCⅡ levels, as corroborated by Western blot analysis. Together, these findings indicate that the p.Arg314Trp variant dually impairs the function of the HCⅡ protein by concurrently reducing its expression and disrupting its normal localization, which constitutes the underlying basis for its functional impairment.

### Bioinformatic prediction of SERPIND1 and KCNH2 protein structures

**Fig. 5:**
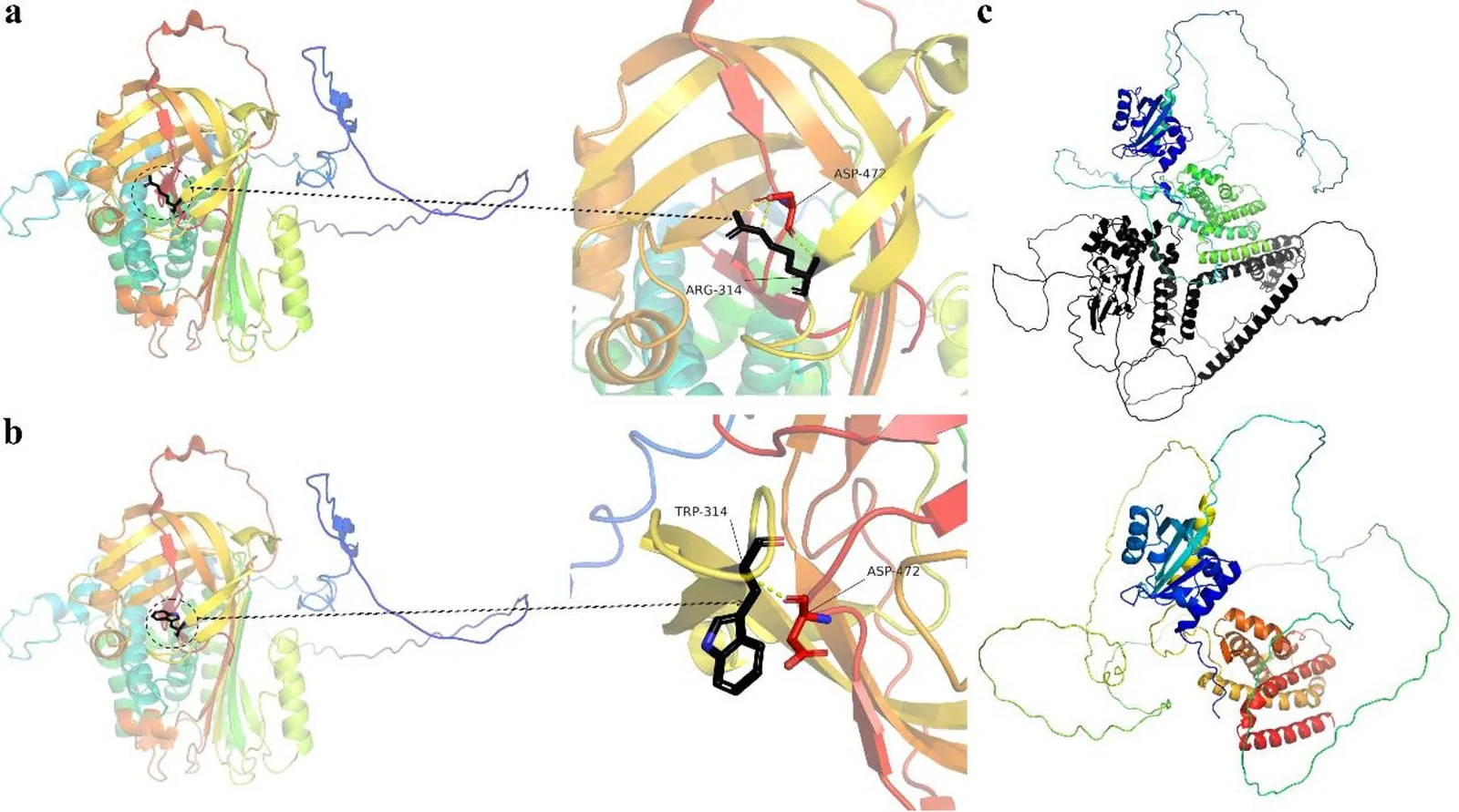
HCⅡ protein (*SERPIND1*) homology modeling and analysis. **a** 3D structure of WT HCⅡ protein. Arg314 (highlighted in black) forms three hydrogen bonds with Asp472. **b** Mutant HCⅡ structure (p.Arg314Trp). Trp314 (highlighted in black) induces significant conformational rearrangement, reducing hydrogen bonding with Asp472 to a single bond. **c** The upper panel shows the WT human Ether-à-go-go-Related Gene potassium channel (hERG) protein (*KCNH2*). The black region indicates the amino acid sequence lost due to the variant. The lower panel displays the truncated protein predicted following the nonsense variant

The three-dimensional structures of the WT and mutant human HCⅡ were predicted using Swiss-Model (https://swissmodel.expasy.org/repository/uniprot/P05546). Visualization in PyMOL revealed structural alterations in the HCⅡ protein (*SERPIND1*) following the p.Arg314Trp variant, which may impair its hydrogen bonding with aspartate at position 472, reducing the original three hydrogen bonds to one (Fig. 5a and b). Similarly, the tertiary structure of the hERG protein (*KCNH2*) was predicted. The black segment represents the amino acid sequence deleted in the mutant protein, demonstrating that nonsense variant could generate a truncated protein potentially compromising KCNH2 function (Fig. 5c).

## Discussions

We herein report a case of splenic vein thrombosis accompanied by multi-site venous thromboses in a patient carrying a pathogenic *SERPIND1* variant that is associated with impaired HCⅡ expression and function. In addition, the patient was found to harbor a concurrent pathogenic *KCNH2* mutation consistent with LQT2. Our functional data support a loss-of-function effect of the *SERPIND1* variant, suggesting a potential contribution to thrombosis susceptibility. While the *KCNH2* mutation accounts for the LQT2 phenotype, its potential contribution to venous thrombosis remains uncertain and warrants further investigation.

Sanyal [24] et al. and Madsen [25] et al. recognized that IGV1 are predominantly caused by splenic vein occlusion or stenosis, often secondary to pancreatic pathologies, while cirrhosis accounts for only 2% of cases. The presence of IGV1 in the absence of pancreatic, hepatic, or other identifiable etiologies should raise suspicion for a systemic prothrombotic condition. Although IGV1 secondary to splenic vein thrombosis have been reported in association with ET, PS deficiency, and APS [6, 7], the etiology of many cases of splenic vein thromboses in clinical practice remain classified as idiopathic, suggesting the possible presence of unidentified hereditary thrombophilic conditions.

HCⅡ mainly expressed in the liver, inhibits plasma thrombin to regulate coagulation and has been linked to thrombosis when deficient [8, 26]. HCⅡ encoded by the *SERPIND1* gene on chromosome 22q11.21, is a serine protease inhibitor that specifically inhibits thrombin [10, 11]. Unlike ATIII which requires activation by heparin or heparan sulfate, HCⅡ is primarily activated by DS, a glycosaminoglycan abundantly present in the vascular extracellular matrix [27–29]. Although in vitro studies have shown that heparin can also activate HCⅡ (Kd=12 μm), its binding affinity and activation efficiency are significantly lower compared to DS (Kd = 0.8 μm) [27]. Following vascular injury, HCⅡ binds to exposed DS, inducing a conformational change that enhances its thrombin-inhibitory activity by more than 1,00-fold [30]. This interaction involves the RCL forming a covalent complex with thrombin, establishing a localized anticoagulant barrier at injury sites [18, 31]. The mature HCⅡ protein consists of 499 amino acids and is functionally organized into four key regions [32]. The N-terminal acidic region (1–75 amino acids), rich in glutamate repeats, specifically binds DS and mediates glycosaminoglycan-dependent activation. The Helix D region (121–145 amino acids) undergoes conformational change to expose the reactive center loop (RCL). The β-sheet A region (200–280 amino acids) forms the serpin structural core and participates in signal transmission. The RCL domain (444–455 amino acids) contains the thrombin recognition and cleavage site at Arg444–Thr445, which is essential for HCⅡ function. Upon binding to thrombin, this region is cleaved, leading to the formation of a stable covalent complex that inactivates thrombin and results in irreversible inhibition [13, 15, 33–37].

However, some studies suggest that it contributes minimally to normal hemostasis, and thus, whether HCⅡ is a definitive risk factor for thrombosis remains inconclusive [38, 39]. We identified a *SERPIND1* c.940 C > T variant in exon 3, resulting in a p.Arg314Trp substitution within the HCⅡ protein structure. This substitution disrupts at least one of the three hydrogen bonds normally formed with aspartic acid 472, thereby inducing conformational changes in HCⅡ (Fig. 5a, b). Our experimental results demonstrated that this variant significantly downregulate *SERPIND1* mRNA levels, as consistently confirmed by q-PCR using primer pairs flanking the variant site (Fig. 3b, c). Consequently, Western blot analysis revealed a marked reduction in HCⅡ protein expression in cells harboring the mutant compared to wild-type (Fig. 3f, g). Immunofluorescence analysis confirmed an altered subcellular localization of the mutant protein (Fig. 4), providing visual evidence of its misfolding and misrouting. This phenomenon likely stems from impaired translational and post-translational modifications caused by the mutant residue, leading to defective protein processing and secretion-a predominant molecular mechanism underlying most missense variants and hereditary disorders [40]. Therefore, the *SERPIND1* c.940 C > T variant impairs HCⅡ function by reducing protein expression and altering its cellular localization. This finding supports a potential role of HCⅡ deficiency in thrombosis susceptibility.

Notably, epidemiological studies have revealed comparable prevalence rates of heterozygous HCⅡ deficiency between the general healthy population and patients with deep vein thrombosis (DVT) [39]. In this familial case, the proband herself experienced two pregnancy-associated DVT episodes. The first thrombotic event manifested as acute left lower limb swelling immediately following her initial delivery, with transient anticoagulation achieving clinical resolution but no subsequent long-term prophylactic management. During her second pregnancy, therapeutic anticoagulation was implemented but discontinued prematurely postpartum. Critically, no post-treatment monitoring protocol was followed, culminating in delayed recognition of splenic vein occlusion and life-threatening gastric variceal hemorrhage. Previous studies have demonstrated that HCⅡ-deficient mice exhibit accelerated thrombus formation following vascular injury, underscoring the essential role of HCⅡ in maintaining vascular homeostasis and regulating hemostatic balance [41]. Likewise, some studies have reported that individuals with reduced plasma HCⅡ levels may not show an elevated risk of venous thrombosis under normal conditions [42], but they appear to be more prone to atherosclerosis or neointimal hyperplasia following angioplasty or stent implantation [43, 44]. Pregnancy is widely recognized as a physiological hypercoagulable state [45]. Notably, the placenta is enriched with DS, and trace amounts of DS are also present in both maternal and fetal plasma [46, 47]. DS acts as a potent cofactor for HCⅡ, promoting its activation and significantly enhancing thrombin inhibition [48]. This DS-mediated activation of HCⅡ constitutes an intrinsic protective mechanism designed to counterbalance the heightened thrombotic risk associated with pregnancy. However, if HCⅡ function is compromised, this compensatory anticoagulant pathway may be insufficient, thereby increasing the likelihood of thrombotic events during or following pregnancy. This clinical trajectory strongly suggests that in female heterozygous carriers, physiological stressors such as the hypercoagulable state of pregnancy or postpartum vascular remodeling may unmask HCⅡ insufficiency, precipitating thrombotic events that remain quiescent under baseline conditions.

The *KCNH2* c.1707 C > A (p.Tyr569*) nonsense mutation introduces a premature termination codon at position 569, resulting in truncated protein synthesis and complete LOF of the K_v_11.1 potassium channel [20]. This LOF manifests clinically as prolonged QT interval on ECG, consistent with LQT2 [49]. No clinical or experimental evidence in this case supports an association between LQT2 and venous thrombosis. Therefore, *KCNH2* mutation is considered an independent and unrelated genetic finding.

This study has several limitations that warrant cautious interpretation. First, the genetic and clinical data were derived from a single family, which inherently limits the ability to establish definitive causality or robust genotype–phenotype correlations. The observed co-segregation pattern does not exclude the influence of additional genetic modifiers or environmental and acquired risk factors. Second, the clinical manifestations among variant carriers were heterogeneous, suggesting possible variable penetrance and expressivity of the variant. Third, whether pregnancy affects HCⅡ function was not experimentally verified. To address this, further in vitro studies using pregnancy-related hormonal stimulation models or animal models of pregnancy could be employed to elucidate the potential modulatory role of the gestational state on HCⅡ activity. Therefore, our findings should be interpreted as preliminary evidence supporting a potential association rather than definitive proof of pathogenicity. Further validation in larger, independent cohorts is required to confirm the role of this variant in thrombosis susceptibility.

## Conclusion

This study identified the heterozygous *SERPIND1* c.940 C > T (p.Arg314Trp) variant, which is associated with impaired HCⅡ expression and abnormal cellular localization. These findings support a loss-of-function effect and suggest a potential role of HCⅡ deficiency in thrombosis susceptibility. Concurrently, a heterozygous *KCNH2* c.1707 C > A (p.Tyr569*) mutation was identified and is consistent with a diagnosis of LQT2. No clinical or experimental evidence in this case supports an association between LQT2 and venous thrombosis; therefore, it is considered an independent and unrelated genetic finding. Overall, the *SERPIND1* variant represents the principal genetic factor associated with the thrombotic phenotype and IGV1 in this family, while *KCNH2* mutation is an incidental finding unrelated to thrombosis.

## Supplementary Information

Supplementary Material 1.

Supplementary Material 2.

## Data Availability

The data underlying the results presented in the study are available from National Center for Biotechnology Information: SCV005908083 (https://www.ncbi.nlm.nih.gov/clinvar/variation/3778884/) and SCV006550964 (https://www.ncbi.nlm.nih.gov/clinvar/variation/4280330/).

## References

[1] Luo X, Hernandez-Gea V. Update on the management of gastric varices [J]. Liver Int. 2022;42(6):1250–8. 10.1111/liv.15181 35129288

[2] Lee EW, Shahrouki P, Alanis L, et al. Management Options for Gastric Variceal Hemorrhage [J]. JAMA Surg. 2019;154(6):540–8. 10.1001/jamasurg.2019.0407 30942880

[3] Simonetto DA, Liu M, Kamath PS. Portal Hypertension and Related Complications: Diagnosis and Management [J]. Mayo Clin Proc. 2019;94(4):714–26. 10.1016/j.mayocp.2018.12.020 30947834

[4] Blei AT. Portal hypertension and its complications [J]. Curr Opin Gastroenterol. 2007;23(3):275–82. 10.1097/MOG.0b013e3280b0841f 17414843

[5] Mayer P, Venkatasamy A, Baumert TF, et al. Left-sided portal hypertension: Update and proposition of management algorithm [J]. J Visc Surg. 2024;161(1):21–32. 10.1016/j.jviscsurg.2023.11.005 38142180

[6] Watanabe Y, Osaki A, Yamazaki S, et al. Two Cases of Gastric Varices with Left-sided Portal Hypertension Due to Essential Thrombocythemia Treated with Gastric Devascularization or Partial Splenic Embolization [J]. Intern Med. 2023;62(19):2839–46. 10.2169/internalmedicine.1273-22 PMC10602822 36823082

[7] Li X, Wu J, Fang F, et al. Isolated gastric varices associated with antiphospholipid syndrome and protein S deficiency: a case report and review of the literature [J]. J Int Med Res. 2024;52(4):3000605241240579. 10.1177/03000605241240579 PMC11010754 38603605

[8] Simioni P. The molecular genetics of familial venous thrombosis [J]. Baillieres Best Pract Res Clin Haematol. 1999;12(3):479–503. 10.1053/beha.1999.0037 10856982

[9] Andriani A, Latagliata R, Anaclerico B, et al. Spleen enlargement is a risk factor for thrombosis in essential thrombocythemia: Evaluation on 1,297 patients [J]. Am J Hematol. 2016;91(3):318–21. 10.1002/ajh.24269 26748894

[10] Blinder MA, Marasa JC, Reynolds CH, et al. Heparin cofactor II: cDNA sequence, chromosome localization, restriction fragment length polymorphism, and expression in Escherichia coli [J]. Biochemistry. 1988;27(2):752–9. 10.1021/bi00402a039 2894851

[11] Ragg H. A new member of the plasma protease inhibitor gene family [J]. Nucleic Acids Res. 1986;14(2):1073–88. 10.1093/nar/14.2.1073 PMC339484 3003690

[12] Cooper DN. The molecular genetics of familial venous thrombosis [J]. Blood Rev. 1991;5(1):55–70. 10.1016/0268-960x(91)90009-2 1674436

[13] Baglin TP, Carrell RW, Church FC, et al. Crystal structures of native and thrombin-complexed heparin cofactor II reveal a multistep allosteric mechanism [J]. Proc Natl Acad Sci U S A. 2002;99(17):11079–84. 10.1073/pnas.162232399 PMC123213 12169660

[14] Liaw PC, Austin RC, Fredenburgh JC, et al. Comparison of heparin- and dermatan sulfate-mediated catalysis of thrombin inactivation by heparin cofactor II [J]. J Biol Chem. 1999;274(39):27597–604. 10.1074/jbc.274.39.27597 10488098

[15] Gettins PG. Serpin structure, mechanism, and function [J]. Chem Rev. 2002;102(12):4751–804. 10.1021/cr010170+ 12475206

[16] Pike RN, Buckle AM, Le Bonniec BF, et al. Control of the coagulation system by serpins. Getting by with a little help from glycosaminoglycans [J]. FEBS J. 2005;272(19):4842–51. 10.1111/j.1742-4658.2005.04880.x 16176258

[17] Tollefsen DM. The interaction of glycosaminoglycans with heparin cofactor II [J]. Ann N Y Acad Sci. 1994;714:21–31. 10.1111/j.1749-6632.1994.tb12027.x 8017769

[18] Tollefsen DM. Insight into the mechanism of action of heparin cofactor II [J]. Thromb Haemost. 1995;74(5):1209–14. 8607096

[19] Rau JC, Mitchell JW, Fortenberry YM, et al. Heparin cofactor II: discovery, properties, and role in controlling vascular homeostasis [J]. Semin Thromb Hemost. 2011;37(4):339–48. 10.1055/s-0031-1276582 21805439

[20] Trudeau MC, Warmke JW, Ganetzky B, et al. HERG, a human inward rectifier in the voltage-gated potassium channel family [J]. Science. 1995;269(5220):92–5. 10.1126/science.7604285 7604285

[21] Zicha S, Moss I, Allen B, et al. Molecular basis of species-specific expression of repolarizing K+ currents in the heart [J]. Am J Physiol Heart Circ Physiol. 2003;285(4):H1641–9. 10.1152/ajpheart.00346.2003 12816752

[22] Singh V, Wagner KT, Williams LG, et al. Knock-in Kcnh2 rabbit model of long QT syndrome type-2, epilepsy, and sudden death [J]. J Transl Med. 2025;23(1):446. 10.1186/s12967-025-06382-w PMC12001650 40234944

[23] Babcock JJ, Li M. hERG channel function: beyond long QT [J]. Acta Pharmacol Sin. 2013;34(3):329–35. 10.1038/aps.2013.6 PMC3587915 23459091

[24] Sanyal AJ, Bosch J, Blei A, et al. Portal hypertension and its complications [J]. Gastroenterology. 2008;134(6):1715–28. 10.1053/j.gastro.2008.03.007 18471549

[25] Madsen MS, Petersen TH, Sommer H. Segmental portal hypertension [J]. Ann Surg. 1986;204(1):72–7. 10.1097/00000658-198607000-00010 PMC1251225 3729585

[26] Kumar A, Bhandari A, Sarde SJ, et al. Genetic variants and evolutionary analyses of heparin cofactor II [J]. Immunobiology. 2014;219(9):713–28. 10.1016/j.imbio.2014.05.003 24950623

[27] Griffith MJ, Marbet GA. Dermatan sulfate and heparin can be fractionated by affinity for heparin cofactor II [J]. Biochem Biophys Res Commun. 1983;112(2):663–70. 10.1016/0006-291x(83)91514-0 6687802

[28] Tollefsen DM. Activation of heparin cofactor II by heparin and dermatan sulfate [J]. Nouv Rev Fr Hematol (1978). 1984;26(4):233-7. 6548014

[29] Church FC, Griffith MJ. Evidence for essential lysines in heparin cofactor II [J]. Biochem Biophys Res Commun. 1984;124(3):745–51. 10.1016/0006-291x(84)91021-0 6439196

[30] Derechin VM, Blinder MA, Tollefsen DM. Substitution of arginine for Leu444 in the reactive site of heparin cofactor II enhances the rate of thrombin inhibition [J]. J Biol Chem. 1990;265(10):5623–8. 2138609

[31] Rau JC, Beaulieu LM, Huntington JA, et al. Serpins in thrombosis, hemostasis and fibrinolysis [J]. J Thromb Haemost. 2007;5(Suppl 1):102–15. 10.1111/j.1538-7836.2007.02516.x PMC2670448 17635716

[32] Law RH, Zhang Q, Mcgowan S, et al. An overview of the serpin superfamily [J]. Genome Biol. 2006;7(5):216. 10.1186/gb-2006-7-5-216 PMC1779521 16737556

[33] Huntington JA, Read RJ, Carrell RW. Structure of a serpin-protease complex shows inhibition by deformation [J]. Nature. 2000;407(6806):923–6. 10.1038/35038119 11057674

[34] Whinna HC, Blinder MA, Szewczyk M, et al. Role of lysine 173 in heparin binding to heparin cofactor II [J]. J Biol Chem. 1991;266(13):8129–35. 1902471

[35] Blinder MA, Tollefsen DM. Site-directed mutagenesis of arginine 103 and lysine 185 in the proposed glycosaminoglycan-binding site of heparin cofactor II [J]. J Biol Chem. 1990;265(1):286–91. 2104620

[36] Mitchell JW, Church FC. Aspartic acid residues 72 and 75 and tyrosine-sulfate 73 of heparin cofactor II promote intramolecular interactions during glycosaminoglycan binding and thrombin inhibition [J]. J Biol Chem. 2002;277(22):19823–30. 10.1074/jbc.M200630200 11856753

[37] Tollefsen DM. Heparin cofactor II modulates the response to vascular injury [J]. Arterioscler Thromb Vasc Biol. 2007;27(3):454–60. 10.1161/01.ATV.0000256471.22437.88 17194895

[38] Toulon P, Moulonguet-Doleris L, Costa JM, et al. Heparin cofactor II deficiency in renal allograft recipients: no correlation with the development of thrombosis [J]. Thromb Haemost. 1991;65(1):20–4. 2024235

[39] Bertina RM, Van Der Linden IK, Engesser L, et al. Hereditary heparin cofactor II deficiency and the risk of development of thrombosis [J]. Thromb Haemost. 1987;57(2):196–200. 3603411

[40] Andersen BD, Bisgaard ML, Lind B, et al. Characterization and structural impact of five novel PROS1 mutations in eleven protein S-deficient families [J]. Thromb Haemost. 2001;86(6):1392–9. 11776305

[41] He L, Giri TK, Vicente CP, et al. Vascular dermatan sulfate regulates the antithrombotic activity of heparin cofactor II [J]. Blood. 2008;111(8):4118–25. 10.1182/blood-2007-12-127928 PMC2288722 18281504

[42] Tollefsen DM. Heparin cofactor II deficiency [J]. Arch Pathol Lab Med. 2002;126(11):1394–400. 10.5858/2002-126-1394-HCID 12421148

[43] Aihara K. Heparin cofactor II attenuates vascular remodeling in humans and mice [J]. Circ J. 2010;74(8):1518–23. 10.1253/circj.cj-10-0577 20671370

[44] Aihara K, Azuma H, Akaike M, et al. Heparin cofactor II as a novel vascular protective factor against atherosclerosis [J]. J Atheroscler Thromb. 2009;16(5):523–31. 10.5551/jat.1552 19729870

[45] Middeldorp S, Nieuwlaat R, Baumann Kreuziger L, et al. American Society of Hematology 2023 guidelines for management of venous thromboembolism: thrombophilia testing [J]. Blood Adv. 2023;7(22):7101–38. 10.1182/bloodadvances.2023010177 PMC10709681 37195076

[46] Achur RN, Valiyaveettil M, Alkhalil A, et al. Characterization of proteoglycans of human placenta and identification of unique chondroitin sulfate proteoglycans of the intervillous spaces that mediate the adherence of Plasmodium falciparum-infected erythrocytes to the placenta [J]. J Biol Chem. 2000;275(51):40344–56. 10.1074/jbc.M006398200 11005814

[47] Andrew M, Mitchell L, Berry L, et al. An anticoagulant dermatan sulfate proteoglycan circulates in the pregnant woman and her fetus [J]. J Clin Invest. 1992;89(1):321–6. 10.1172/JCI115579 PMC442851 1729278

[48] Giri TK, Tollefsen DM. Placental dermatan sulfate: isolation, anticoagulant activity, and association with heparin cofactor II [J]. Blood. 2006;107(7):2753–8. 10.1182/blood-2005-09-3755 PMC1895383 16339402

[49] Schwartz PJ, Ackerman MJ, Antzelevitch C, et al. Inherited cardiac arrhythmias [J]. Nat Rev Dis Primers. 2020;6(1):58. 10.1038/s41572-020-0188-7 PMC7935690 32678103

