# Supplementary material for: Genetic and functional characterization of a novel SERPIND1 variant in a family with isolated gastric varices and venous thrombosis
Source: BMC Gastroenterol. 2026 Jul 23;26:672. doi: 10.1186/s12876-026-05163-z (PMC13682084; doi:10.1186/s12876-026-05163-z)
Supplement: Supplementary file 1 [file 12876_2026_5163_MOESM2_ESM.docx]

**Supplementary Table 1** Laboratory evaluation for inherited and acquired thrombophilia in the proband.

| **Test item** | **Result** | **Reference value** |
| --- | --- | --- |
| HCII antigen (ng/mL) | 65 | 80-120 |
| HCII activity (%) | 48 | 70-120 |
| Lupus anticoagulant tests LA1 (U/ml) | 37.2 | 31.0-44.0 |
| Lupus anticoagulant tests LA2 (U/ml) | 33.7 | 30.0-38.0 |
| Anticardiolipin antibodies IgG (sec) | 3.2 | <8.0, negtive |
| Anticardiolipin antibodies IgM (sec) | 2.9 | <8.0, negtive |
| Homocysteine（umol/L） | 8.5 | 5.0-15.0 |

Note: HCII heparin cofactor II.

**Supplementary Table 2** Calculation of WB gray value.

| **Group** | **Lane** | **GAPDH** | **HA tag** | **HA tag/GAPDH** |
| --- | --- | --- | --- | --- |
| Control | 1 | 35225.045 | 644.092 | 0.018285058 |
|  | 2 | 44370.368 | 119.607 | 0.00269565 |
|  | 3 | 43832.125 | 1564.062 | 0.035683006 |
| SERPIND1-MUT | 1 | 41248.004 | 13837.832 | 0.335478827 |
|  | 2 | 54353.196 | 11947.468 | 0.219811692 |
|  | 3 | 57815.459 | 9662.903 | 0.167133552 |
| SERPIND1-WT | 1 | 50269.731 | 37089.51 | 0.737809995 |
|  | 2 | 53706.61 | 36218.681 | 0.674380323 |
|  | 3 | 49980.752 | 38737.731 | 0.775052984 |

**Supplementary Table 3** Genotype–phenotype correlation and clinical characteristics of family members.

| **Individual** | **Relationship** | **Age**  **(years)** | **Sex** | ***SERPIND1* c.940C>T** | ***KCNH2* c.1707C>A** | **IGV1** | **LQT2** | **Thrombosis history** | **Thrombosis assessment** | **HCII antigen (ng/mL)** | **HCII activity (%)** | **Anticoagulation at sampling** | **Other risk factors** |
| --- | --- | --- | --- | --- | --- | --- | --- | --- | --- | --- | --- | --- | --- |
| I-1 | Father | 62 | M | WT | Het | No | Yes | No | Ultrasound | 110 | 112 | No | None |
| I-2 | Mother | 60 | F | WT | WT | No | No | No | Ultrasound | 100 | 102 | No | None |
| II-1 | Brother | 36 | M | WT | WT | No | No | No | Ultrasound | 102 | 106 | No | None |
| II-2 | Brother | 34 | M | WT | WT | No | No | No | Ultrasound | 101 | 104 | No | None |
| II-4 | Proband | 31 | F | Het | Het | Yes | Yes | Yes (DVT) | CT/MRI/  DSA | 65 | 48 | No | Postpartum state |
| III-3 | Daughter | 8 | F | Het | Het | Not assessed | Yes | Yes (DVT) | Ultrasound | 70 | 52 | No | None |
| III-4 | Daughter | 5 | F | WT | WT | Not assessed | No | No | Ultrasound | 99 | 101 | No | None |

Note: IGV1 Isolated Gastric Varices Type 1; LQT2 Long QT Syndrome Type 2; HCII Heparin cofactor II; M Male; F Female; WT Wild type; Het Heterozygous; DVT Deep Vein Thrombosis; CT Computed Tomography; MRI Magnetic Resonance Imaging; DSA Digital Subtraction Angiography; HCII antigen reference range: 80–120 ng/mL; HCII activity reference range: 70–120%.
