# Supplementary material for: Genetic and functional characterization of a novel SERPIND1 variant in a family with isolated gastric varices and venous thrombosis
Source: BMC Gastroenterol. 2026 Jul 23;26:672. doi: 10.1186/s12876-026-05163-z (PMC13682084; doi:10.1186/s12876-026-05163-z)
Supplement: Supplementary file 2 [file 12876_2026_5163_MOESM1_ESM.zip › Supplementary file 1/Supplementary File 1 – Raw Blots Information.doc]

Supplementary File 1–Raw Blots Information

This folder contains all full-length, original, and unprocessed Western blot images corresponding to the figures presented in the manuscript:

| Figure | Protein | File name | Description |
| --- | --- | --- | --- |
| Figure 3(f) | GAPDH | Figure3F_GAPDH_raw.tif | Full-length original blot for GAPDH used in Figure 3(f) |
| Figure 3(f) | HA tag | Figure3F_HA tag_raw.tif | Full-length original blot for HA tag used in Figure 3(f) |

Notes:

* All blots show visible membrane edges and ladder markings where available.

* Images are unprocessed except for uniform contrast adjustment applied to the entire image.

* The blots are directly derived from the original experimental records.
